# *In Amnio* MRI of Mouse Embryos

**DOI:** 10.1371/journal.pone.0109143

**Published:** 2014-10-15

**Authors:** Thomas A. Roberts, Francesca C. Norris, Helen Carnaghan, Dawn Savery, Jack A. Wells, Bernard Siow, Peter J. Scambler, Agostino Pierro, Paolo De Coppi, Simon Eaton, Mark F. Lythgoe

**Affiliations:** 1 UCL Centre for Advanced Biomedical Imaging, Division of Medicine, University College London, London, United Kingdom; 2 UCL Centre for Mathematics and Physics in the Life Sciences and Experimental Biology, University College London, London, United Kingdom; 3 UCL Institute of Child Health, University College London, London, United Kingdom; University of Washington School of Medicine, United States of America

## Abstract

Mouse embryo imaging is conventionally carried out on *ex vivo* embryos excised from the amniotic sac, omitting vital structures and abnormalities external to the body. Here, we present an *in amnio* MR imaging methodology in which the mouse embryo is retained in the amniotic sac and demonstrate how important embryonic structures can be visualised in 3D with high spatial resolution (100 µm/px). To illustrate the utility of *in amnio* imaging, we subsequently apply the technique to examine abnormal mouse embryos with abdominal wall defects. Mouse embryos at E17.5 were imaged and compared, including three normal phenotype embryos, an abnormal embryo with a clear exomphalos defect, and one with a suspected gastroschisis phenotype. Embryos were excised from the mother ensuring the amnion remained intact and stereo microscopy was performed. Embryos were next embedded in agarose for 3D, high resolution MRI on a 9.4T scanner. Identification of the abnormal embryo phenotypes was not possible using stereo microscopy or conventional *ex vivo* MRI. Using *in amnio* MRI, we determined that the abnormal embryos had an exomphalos phenotype with varying severities. *In amnio* MRI is ideally suited to investigate the complex relationship between embryo and amnion, together with screening for other abnormalities located outside of the mouse embryo, providing a valuable complement to histology and existing imaging methods available to the phenotyping community.

## Introduction

Mice are widely used as research models for investigating development and disease as the murine genome can be readily manipulated to create phenotypes analogous to human conditions. Large-scale international programmes are underway to generate knockout mice for each of the approximate 23,000 protein-encoding genes in the mouse genome [1]. The broad aim is to build a library of gene function, which will facilitate research into human diseases and the development of new drugs and therapies [2]. Mouse embryo phenotyping will be an important part of this challenge since an estimated 30% of all targeted genes will lead to intrauterine lethality when inactivated or perinatal demise [3], which will preclude analysis of these genes in adult mice, unless conditional mutagenesis is used for investigating tissue-specific gene function [4].

A host of advanced imaging technologies exist for characterising the developing mouse [5]. Magnetic resonance imaging (MRI) is a well-established technique for monitoring embryonic development and phenotype classification [6] owing to its non-invasive and high-throughput capabilities [7], high resolution (<20 µm) [8] with excellent soft tissue contrast, and the availability of automated computational methods for image analysis [9]. Conventionally, *ex vivo* mouse embryo MRI is carried out on mid to late gestation embryos (typically aged between E14.5 and 18.5), which have been extracted from the mother, removed from the amniotic sac and exsanguinated by cutting the umbilical cord close to the abdomen [10]. Whilst this protocol is sufficient for screening the majority of abnormal embryos, abnormalities associated with the amnion, the placenta or external structures that are fine or easily damaged could be missed. These include placental defects, such as preeclampsia [11] and intrauterine growth retardation [12], as well as abdominal wall abnormalities, such as exomphalos and gastroschisis, in which the defect may not be accurately delineated due to disruption of structures whilst extracting and preparing the embryo [13].

In this study, we describe a new *in amnio* MR imaging method in which the mouse embryo is retained within the amniotic sac, therefore complementing the existing *ex vivo* embryo screening protocol by providing additional information on the developmental relationship between the embryo, fine external structures, umbilical cord and placenta. As a proof of principle, we apply this technique to image E17.5 floxed Scribble (Scrib^fl/-^) heterozygote embryos [14] with abdominal wall defects. Phenotype penetrance was low in the animal model, thus alongside normal phenotype embryos, we examined: an abnormal embryo with a defined exomphalos abnormality, where the herniated abdominal contents protrude into the base of the umbilical cord and are enclosed within a membrane; and an abnormal embryo with a suspected gastroschisis abnormality, where the eviscerated abdominal contents have no covering membrane and are exposed directly to the amniotic fluid.

The aim of the present report was two-fold: to demonstrate the additional information gained imaging *in amnio* and to non-destructively characterise the phenotypes of the abnormal embryos. Phenotype identification was not possible using conventional *ex vivo* embryo MRI, light microscopy or histology as the delicate membranous structures, which were in close proximity of the abdominal contents and amniotic membrane, were damaged during the necessary preparation for these approaches.

## Materials and Methods

### Ethics statement

All animal studies were approved by the University College London Biological Services Ethical Review Committee and licensed under the UK Home Office regulations and the Guidance for the Operation of Animals (Scientific Procedures) Act 1986 (Home Office, London, United Kingdom).

### *In amnio* embryo preparation and optimisation

Dams from one successfully timed mating of floxed Scribble (Scrib^fl/-^) heterozygotes were sacrificed by Schedule 1 cervical dislocation methods at 17.5 days of pregnancy (E17.5). After death was confirmed, the embryos from two litters were carefully removed from the uterus ensuring that the amnion remained intact. Five embryos were identified for imaging by visual assessment under a stereo microscope (Zeiss SV6, USA) including three with a normal phenotype, one abnormal embryo with a known exomphalos (exom) defect (tightly packaged abdominal viscera), and one abnormal embryo with a suspected gastroschisis (s.gas) abnormality (dispersed abdominal viscera). Each embryo was placed in a Petri dish filled with phosphate buffered saline (PBS) and photographed using the stereo microscope. Embryos were then immersed in 4% paraformaldehyde for approximately one hour. For MR imaging, each embryo was carefully embedded in 1% agarose gel within individual 50ml centrifuge tubes to maintain amnion integrity. Embryos were scanned less than 24 hours following extraction and fixation. Samples were stored at 4°C before and after imaging.

Preliminary investigations of the sample preparation indicated that standard (15 ml) centrifuge tubes were too narrow, which caused the fragile amniotic sac to split. Furthermore, it was not possible to stabilise the sac using gauze and immersing the embryo in Fomblin (Galden Perfluorosolv-1), as in some standard embryo protocols [15], due to rupturing of the sac. Finally, we found that attempts to enhance embryo tissue contrast by injecting a gadolinium-based MRI contrast agent (Magnevist, Bayer-Schering, Newbury, UK) directly into the embryo also caused the amniotic sac to collapse.

### MR image acquisition and analysis

Embryos were scanned using a 9.4T VNMRS system (Agilent Technologies, Inc., Santa Clara, CA, USA) with a 33mm volume coil (RAPID Biomedical GmbH, Germany). T2-weighted images were acquired using a 3D Fast Spin Echo sequence with repetition time (TR) = 1500ms, effective echo time (ETE) = 80ms, echo train length (ETL) = 8, echo spacing (ESP) = 20ms, k_0_ = 4 and 3 averages. The matrix size was 256×256×256px with a 25.6×25.6×25.6mm field-of-view (FOV), giving an isotropic resolution of 100 µm/pixel (px), and the total scan time was approximately 10 hours.

Images were converted into the Analyze 7.5 data format using ImageJ (NIH, USA) and visualised using Amira 5.4 (Visage Imaging, Inc., CA, USA). Contrast levels were windowed equally across images being compared. The signal-to-noise (SNR) ratio was measured in the brain, liver, amniotic fluid and agarose using MATLAB (Mathworks Inc., MA, USA) by taking the ratio of the average signal from the region of interest and the standard deviation from a region of background noise (SNR = Signal/Noise SD). The contrast-to-noise ratio (CNR = Signal_1_ – Signal_2_/Noise SD) was also calculated and measured relative to agarose.

## Results

### Stereo microscope images

**Figure 1:**
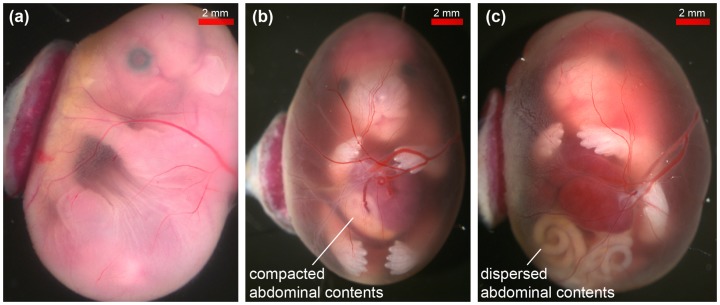
Stereo microscope images of *ex vivo* mouse embryos in their amniotic sacs. (a) normal, (b) known exomphalos, (c) suspected gastroschisis phenotypes. The two abnormal embryos have similar abdominal wall defects but the herninated viscera of the embryo in (c) are much more dispersed than the tightly compacted herniation of the known exomphalos embryo in (b).

The three different types of embryo studied were distinguishable in the stereo microscope images (Figure 1). As expected, the normal phenotype embryos had a complete abdominal wall (Figure 1a), whilst the herniated abdominal contents of the known exomphalos were tightly enclosed in a membrane (Figure 1b). The herniated abdominal contents of the suspected gastroschisis were dispersed, appeared to be exposed to the amniotic fluid (Figure 1c), and were associated with a more extensive abdominal wall defect. Abnormal embryos also exhibited craniorachischisis (Figure S1), which is complete failure of the neural tube to close along the entirety of the cranium and spinal cord. Furthermore, we noted extravasation of blood into the amniotic fluid of both abnormal embryos (Figures 1b and 1c).

### MR imaging optimisation

**Figure 2:**
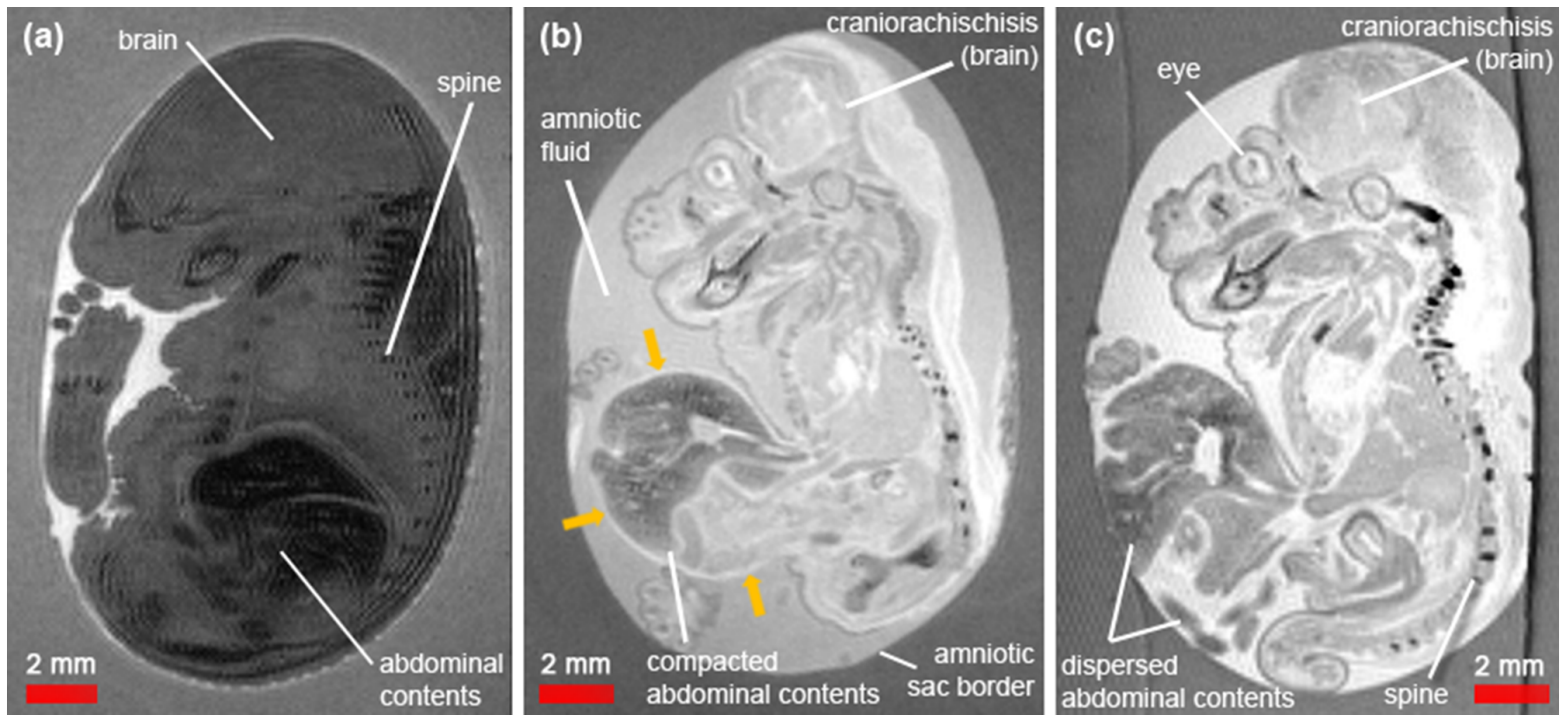
Sagittal sections of *in amnio* mouse embryo MR images at E17.5. (a) normal phenotype, (b) known exomphalos embryo with craniorachischisis and (c) suspected gastroschisis embryo with craniorachischisis. In the exomphalos embryo, the herniated abdominal contents are enclosed in a membrane (yellow arrows) whereas in the suspected gastroschisis they appear exposed to the amniotic fluid.

**Figure 3:**
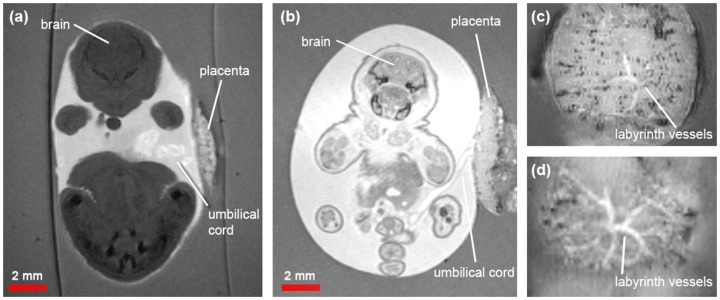
Coronal sections of *in amnio* mouse embryo MR images at E17.5. (a) Normal and (b) known exomphalos embryos: the umbilical cord is visible connecting from the abdomen to the placenta. (c) and (d): Sections through the placenta of the normal and known exomphalos embryos, respectively, showing clearly the labyrinth blood vessels.

Prior experiments investigated the use of a gradient echo sequence. However this gave rise to marked susceptibility artefacts that masked the fine structures of interest due to the blood in the amniotic fluid. For this reason, a T2-weighted 3D Fast Spin Echo sequence was chosen, which was well suited to assess structures of interest in the abnormal embryos as the amniotic fluid provided natural contrast against the embryo tissue and amniotic membrane (Figure 2 and 3).

### *In amnio* MR images

#### Structures visualised with *in amnio* imaging

In addition to the internal organs, which can be imaged with conventional *ex vivo* protocols, important developmental structures could be observed *in amnio,* such as the placenta, umbilical cord and amniotic sac (Figure 3). The hyperintense amniotic fluid was distinct against the hypointense agarose, enabling delineation of the border of the amniotic sac and cross-sections through the embryo placentas showed the labyrinth blood vessels (Figure 3a and 3b). In the abnormal embryos only, we also observed complete failure of the cranium and vertebrae to fuse resulting in externalisation of the brain and spinal cord (Figures 2b and 2c), which matched the observations of craniorachischisis made with light microscopy.

#### Signal disparity between normal phenotypes and abnormal embryos

A marked signal intensity difference was observed between the normal phenotype and abnormal embryos (Figures 2 and 3). For example, the abnormal embryos exhibited a positive CNR in the brain and liver (relative to the agarose) compared to the normal embryos which all had a negative CNR (Table 1). The abnormal embryos exhibited at least a three-fold higher SNR in the brain (SNR_norm_ = 19.5±3.2 vs. SNR_exom_ = 75.9 and SNR_s.gas_ = 66.7) and nine-fold higher SNR in the liver (SNR_norm_ = 5.3±0.9 vs. SNR_exom_ = 46.7, SNR_s.gas_ = 44.9). Conversely, the agarose signal was relatively constant across all embryos (SNR_norm_ = 29.9±0.4 vs. SNR_exom_ = 35.1 and SNR_s.gas_ = 30.2) as was the amniotic fluid signal (SNR_norm_ = 63.1±2.3, SNR_exom_ = 73.3, SNR_s.gas_ = 74.6).

**Table 1:** Summary of SNR and CNR measurements in regions of interest within the embryos (Norm values±SEM).

Embryo	SNR brain	SNR liver	SNR amniotic fluid	SNR agarose	CNR brain-agarose	CNR liver-agarose	CNR amniotic fluid-agarose
**Norm (n = 3)**	19.5±3.2	5.3±0.9	63.1±2.3	29.9±0.4	-10.4±3.4	-24.6±1.1	33.2±2.1
**Exom**	75.9	46.7	73.3	35.1	40.8	11.6	38.2
**S.Gas**	66.7	44.9	74.6	30.2	36.5	14.7	44.4

#### Phenotyping study: exomphalos or gastroschisis?

**Figure 4:**
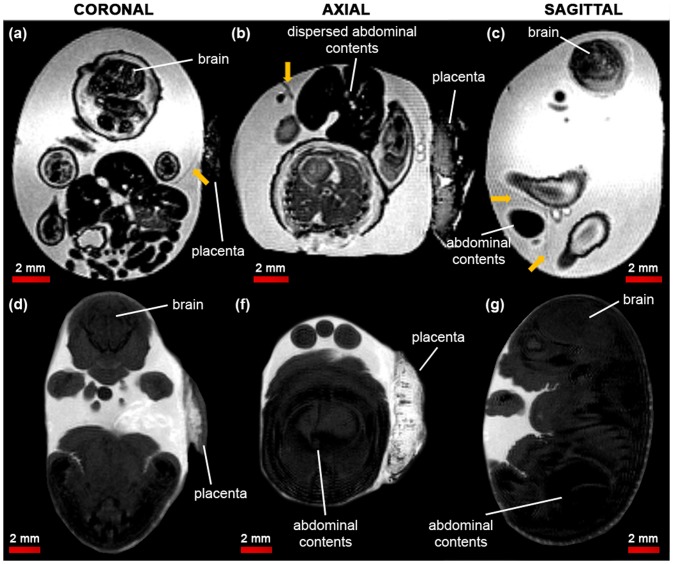
Contrast-adjusted *in amnio* MR images of the normal and suspected gastroschisis embryos at E17.5. (a) coronal, (b) axial and (c) sagittal sections through the suspected gastroschisis mouse embryo. Thin membranous structures (yellow arrows) can be seen extending outwards from the abdomen of the mouse embryo. (d), (e) and (f) show equivalent images of a normal embryo, where the abdominal contents are internalised and no emanating membranes can be observed.

Using our *in amnio* imaging method, we confirmed that the viscera of the known exomphalos embryo were clearly enclosed within a membrane (yellow arrows in Figure 2b), which could be traced back to the abdomen. Furthermore, we determined that the suspected gastroschisis embryo had an exomphalos defect rather than a true gastroschisis defect. Whilst the stereo microscope image (Figure 1c) showed that the herniated abdominal contents were dispersed and may have been exposed to the amniotic fluid, the MR images (Figure 4a, 4b, and 4c) revealed thin (<200 µm), dark structures extending outwards from the abdomen of the embryo, which appear to be remnants of a ruptured membrane. These membranous structures were not present in the normal embryos (Figure 4d, 4e, and 4f).

## Discussion

In this study, we have developed an *in amnio* MRI method for imaging *ex vivo* mouse embryos retained within their amniotic sac. As a proof of principle, we applied our technique to phenotyping a mouse model with an ambiguous abdominal wall abnormality, and determined that it exhibits an exomphalos defect of varying severity.

Whilst other animal models have been studied *in ovo* [16] using MRI, such as chick embryos, *ex vivo* imaging of mouse embryos is generally conducted on subjects excised from the amniotic sac [5]. *In utero* MRI of mouse embryos is possible, but resolution is limited to the level of whole organs [6] and major vasculature [17] Therefore, this approach can only be used to examine the most obvious phenotypes [18]. *In amnio* MRI facilitates high-resolution information about structures crucial to embryonic development, such as the amniotic sac, placenta and umbilical cord, which would otherwise be unattainable using conventional *ex vivo* approaches or *in utero* MRI. *In amnio* imaging is particularly applicable to developmental studies of the placenta as small structures such as the labyrinth blood vessels can be resolved. With further optimisation of the sequence to increase resolution, such as a smaller field-of-view, or by using diffusion tensor imaging, *in amnio* imaging may be used to examine substructures in the placenta.

Optimisation of the embryo preparation determined that embedding in agarose was suitable for *in amnio* imaging without damaging the delicate amniotic sac, as in conventional ex vivo mouse embryo imaging protocols. One hour fixation was shorter than in other protocols [15], however this appeared sufficient for imaging the fine external structures, which remained intact. *In amnio* imaging is compatible with multi-embryo phenotyping protocols [19] as it is possible to embed up to six embryos in agarose at once, depending on gestational age. A multiple embryo study was difficult to conduct in the present work as the phenotype penetrance was low. One limitation of the *in amnio* preparation is that tissue samples cannot be extracted directly from the embryos before imaging as the amniotic sac must be compromised. Alternatively, there are methods for recovering DNA from fixed samples [20] or careful extraction of tissue from the placenta could be conducted before fixation and embedding.

Scan duration was approximately 10 hours, which was convenient for imaging overnight, however acquisition time could be shortened by using a smaller field-of-view or 2D imaging if whole embryo coverage is not required.

We found that the abnormal embryos had positive contrast and higher SNR than the normal phenotype embryos (Figure 3). This effect was most likely caused by blood extravasation [21] (Figures 1b and 1c) from blood vessels supplying the externalised (not covered by normal tissues such as skin) and exposed brain and spinal cord, as these organs were in direct contact with the irritant amniotic fluid leading to leaky vasculature. The contrast observed in normal phenotype embryos was consistent between litters. Further investigation of the signal disparity was not performed as the change in MR signal did not impact on delineating the gut associated membranes, however a combination T1 and T2 mapping may inform the mechanism of the signal disparity.

Using our *in amnio* imaging method, we confirmed that the herniated abdominal contents of the known exomphalos embryo were fully enclosed in a membrane, and determined that the suspected gastroschisis was also an exomphalos phenotype but with a ruptured membrane. The cause of the rupture was unclear. However, it may have split due to environmental factors such as rubbing against the amniotic sac, or could be due to the natural embryonic growth rate variation *in utero* [22]. This demonstrates the potential of *in amnio* imaging over other MRI techniques, such as a conventional *ex vivo* embryo MRI, in which the embryo preparation would have disrupted the thin membrane covering the abdominal contents, and *in utero* MRI, which would not have been able to resolve such small structures. *In amnio* MRI could easily be extended to examine different defects such as in mouse models of placental development [23], [24]. Furthermore, as *in amnio* imaging provides information unattainable using conventional phenotyping methods, such as histology, it could prove a valuable complement to the embryo screening toolkit by facilitating the formation of a more detailed and complete phenotyping library.

In conclusion, we present the first *in amnio* MRI mouse embryo images for the purposes of phenotyping. Important embryonic structures such as the placenta, umbilical cord and amniotic sac can be visualised *in amnio*. In this paper, we applied the technique to examine abnormal mice with abdominal defects, determining that both had an exomphalos phenotype with different levels of severity. Distinguishing between these subtly different defects would have been extremely difficult with existing imaging methods.

## Supporting Information

Figure S1 **Alternative stereo microscope image showing craniorachischisis in the exomphalos embryo.**
(TIF)

Video S1 **3D *in amnio* MRI of a wild-type embryo.**
(AVI)

Video S2 **3D *in amnio* MRI of an exomphalos embryo.**
(AVI)

Video S3 **3D *in amnio* MRI of a suspected gastroschisis embryo.**
(AVI)
