# Supplementary figures and images for: In Amnio MRI of Mouse Embryos
Source: PLoS One. 2014 Oct 15;9(10):e109143. doi: 10.1371/journal.pone.0109143 (PMC4198080; doi:10.1371/journal.pone.0109143)

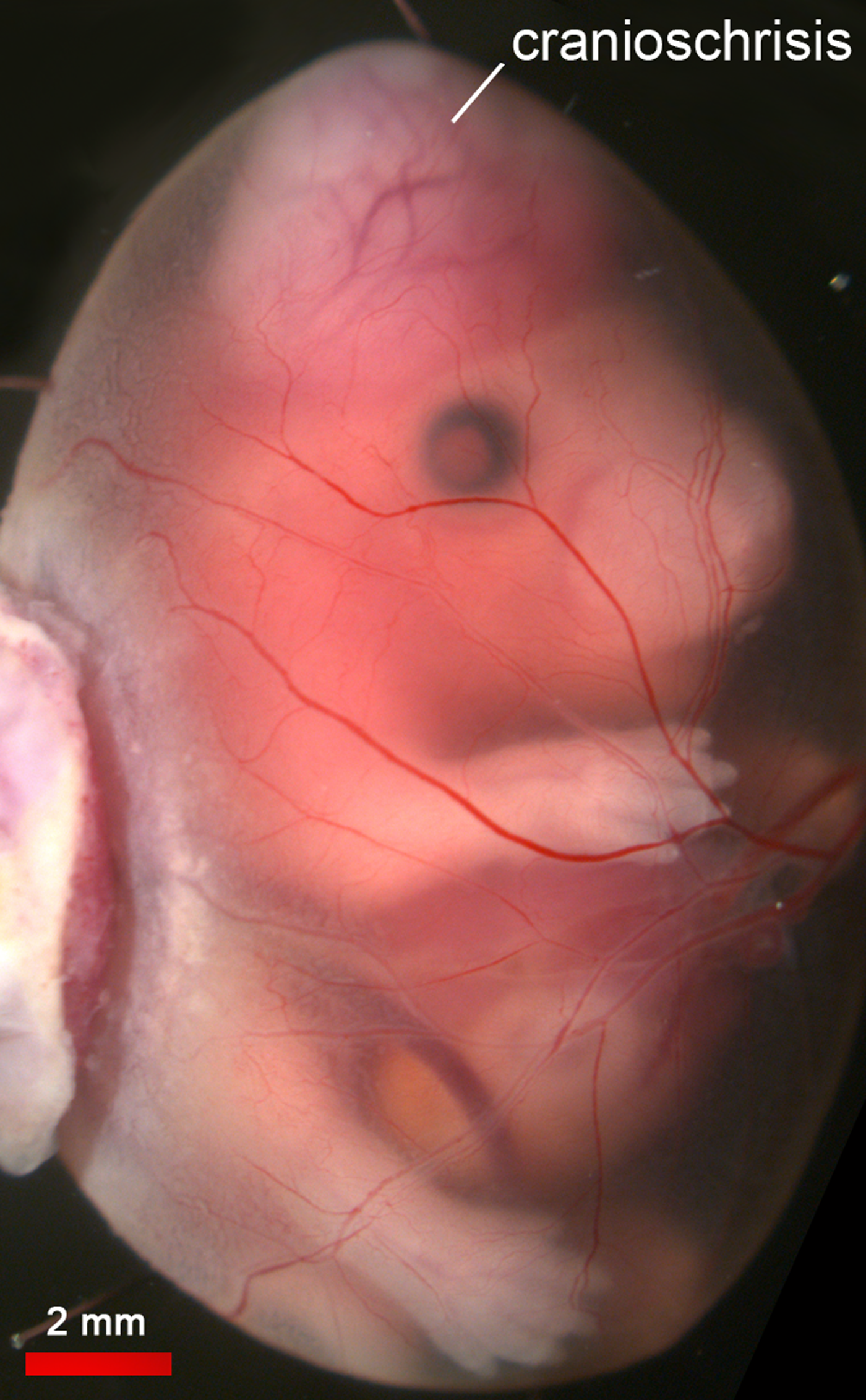

Supplement: Figure S1 — Alternative stereo microscope image showing craniorachischisis in the exomphalos embryo. (TIF) [file pone.0109143.s001.tif]
